# Preclinical and clinical evidence for suppression of alcohol intake by apremilast

**DOI:** 10.1172/JCI159103

**Published:** 2023-03-15

**Authors:** Kolter B. Grigsby, Regina A. Mangieri, Amanda J. Roberts, Marcelo F. Lopez, Evan J. Firsick, Kayla G. Townsley, Alan Beneze, Jessica Bess, Toby K. Eisenstein, Joseph J. Meissler, John M. Light, Jenny Miller, Susan Quello, Farhad Shadan, Michael Skinner, Heather C. Aziz, Pamela Metten, Richard A. Morrisett, John C. Crabbe, Marisa Roberto, Howard C. Becker, Barbara J. Mason, Angela R. Ozburn

**Affiliations:** 1 Portland Alcohol Research Center, Department of Behavioral Neuroscience, Oregon Health & Science University, and VA Portland Health Care System, Portland, Oregon, USA.; 2 Waggoner Center for Alcohol and Addiction Research, Division of Pharmacology and Toxicology, College of Pharmacy, The University of Texas at Austin, Austin, Texas, USA.; 3 Animal Models Core Facility, The Scripps Research Institute, La Jolla, California, USA.; 4 Charleston Alcohol Research Center, Department of Psychiatry and Behavioral Sciences, Medical University of South Carolina, Charleston, South Carolina, USA.; 5 Pearson Center for Alcoholism and Addiction Research, Department of Molecular Medicine, The Scripps Research Institute, La Jolla, California, USA.; 6 Center for Substance Abuse Research, Lewis Katz School of Medicine at Temple University, Philadelphia, Pennsylvania, USA.; 7 Oregon Research Institute, Eugene, Oregon, USA.; 8 Department of Neuroscience, Medical University of South Carolina, Charleston, South Carolina, USA.; 9 RHJ Department of Veterans Affairs Medical Center, Charleston, South Carolina, USA.

**Keywords:** Clinical Trials, Neuroscience, Addiction, Drug therapy

## Abstract

Treatment options for alcohol use disorders (AUDs) have minimally advanced since 2004, while the annual deaths and economic toll have increased alarmingly. Phosphodiesterase type 4 (PDE4) is associated with alcohol and nicotine dependence. PDE4 inhibitors were identified as a potential AUD treatment using a bioinformatics approach. We prioritized a newer PDE4 inhibitor, apremilast, as ideal for repurposing (i.e., FDA approved for psoriasis, low incidence of adverse events, excellent safety profile) and tested it using multiple animal strains and models, as well as in a human phase IIa study. We found that apremilast reduced binge-like alcohol intake and behavioral measures of alcohol motivation in mouse models of genetic risk for drinking to intoxication. Apremilast also reduced excessive alcohol drinking in models of stress-facilitated drinking and alcohol dependence. Using site-directed drug infusions and electrophysiology, we uncovered that apremilast may act to lessen drinking in mice by increasing neural activity in the nucleus accumbens, a key brain region in the regulation of alcohol intake. Importantly, apremilast (90 mg/d) reduced excessive drinking in non–treatment-seeking individuals with AUD in a double-blind, placebo-controlled study. These results demonstrate that apremilast suppresses excessive alcohol drinking across the spectrum of AUD severity.

## Introduction

Alcohol use disorder (AUD) is a complex psychiatric disease with far reaching impacts on society, including more than 95,000 associated deaths annually in the United States and a net economic cost of $249 billion annually (or $807/US individual) (1). Despite growing knowledge of important genetic and molecular mechanisms, pharmacological treatment options for AUD have only minimally advanced since the US Food and Drug Administration (FDA) approval of acamprosate in 2004 (2, 3). Substantial work supports immune and inflammatory pathways as critical regulators of AUDs at all stages of the disease, namely binge drinking, high motivation to drink, and alcohol dependence (4–6). In particular, cyclic adenosine monophosphate–specific (cAMP-specific) phosphodiesterase type 4 (PDE4) has been associated with both alcohol and nicotine dependence in a genome-wide association study and has gained recent attention as a potential molecular target for treating AUD (7).

A central goal of the present study was to determine whether apremilast reduced ethanol drinking across the progression of AUD by testing its efficacy in relevant preclinical drinking paradigms, genetic animal models, and human participants. Specifically, the effects of apremilast were evaluated in 5 clinically relevant animal models of excessive alcohol drinking (listed in order of increasing chronicity): (a) binge-like drinking (8), (b) motivation for self-administration (9), (c) drinking despite negative consequences (a model of compulsive-like alcohol drinking) (10–12), (d) stress-facilitated escalation of drinking (13), and (e) dependence-induced escalation of drinking (14, 15). To complement and extend our preclinical behavioral genetics and pharmacology studies, a double-blind, placebo controlled clinical proof-of-concept (POC) study was conducted to determine the effects of apremilast in non–treatment-seeking individuals with AUD.

We further studied the role of PDE4 in the nucleus accumbens (NAc) in drinking behavior and physiology using genetic mouse models. An extensive body of literature supports the NAc as a critical regulator of alcohol drinking and its candidacy as a neural target in the treatment of AUD (16–21). Therefore, we sought to determine (a) whether administration of apremilast into the NAc would be sufficient to reduce binge-like drinking and achieved blood alcohol levels and (b) whether apremilast differentially alters physiology in 2 types of medium spiny neurons (MSNs; dopamine receptor D1– or D2–expressing MSNs), which comprise the 2 major output pathways from the NAc. Taken together, these studies provide an integrative and rigorous framework supporting further testing of the importance of apremilast as a pharmacotherapy in the treatment of AUDs.

## Results

### Apremilast reduces binge-like drinking behavior and the motivation for ethanol in a genetic risk model of drinking to intoxication.

To test whether PDE4 inhibition reduces binge-like alcohol drinking, we administered apremilast to selectively bred “High Drinking in the Dark” (replicate HDID-1 and HDID-2) mice of both sexes prior to measuring limited access drinking using the widely adopted “Drinking in the Dark” (DID) assay (8). HDID mice reliably reach blood alcohol levels (BALs) well over pharmacological intoxication (defined as 80 mg% — corresponding to the legal level of intoxication; ref. 22). The PDE4 inhibitor rolipram was shown to reduce binge-like ethanol intake and BALs in female and male HDID-1 at all 3 doses tested: 5, 7.5, and 10 mg/kg (i.p.) (Supplemental Figure 1, A and B; supplemental material available online with this article; https://doi.org/10.1172/JCI159103DS1).

**Figure 1:**
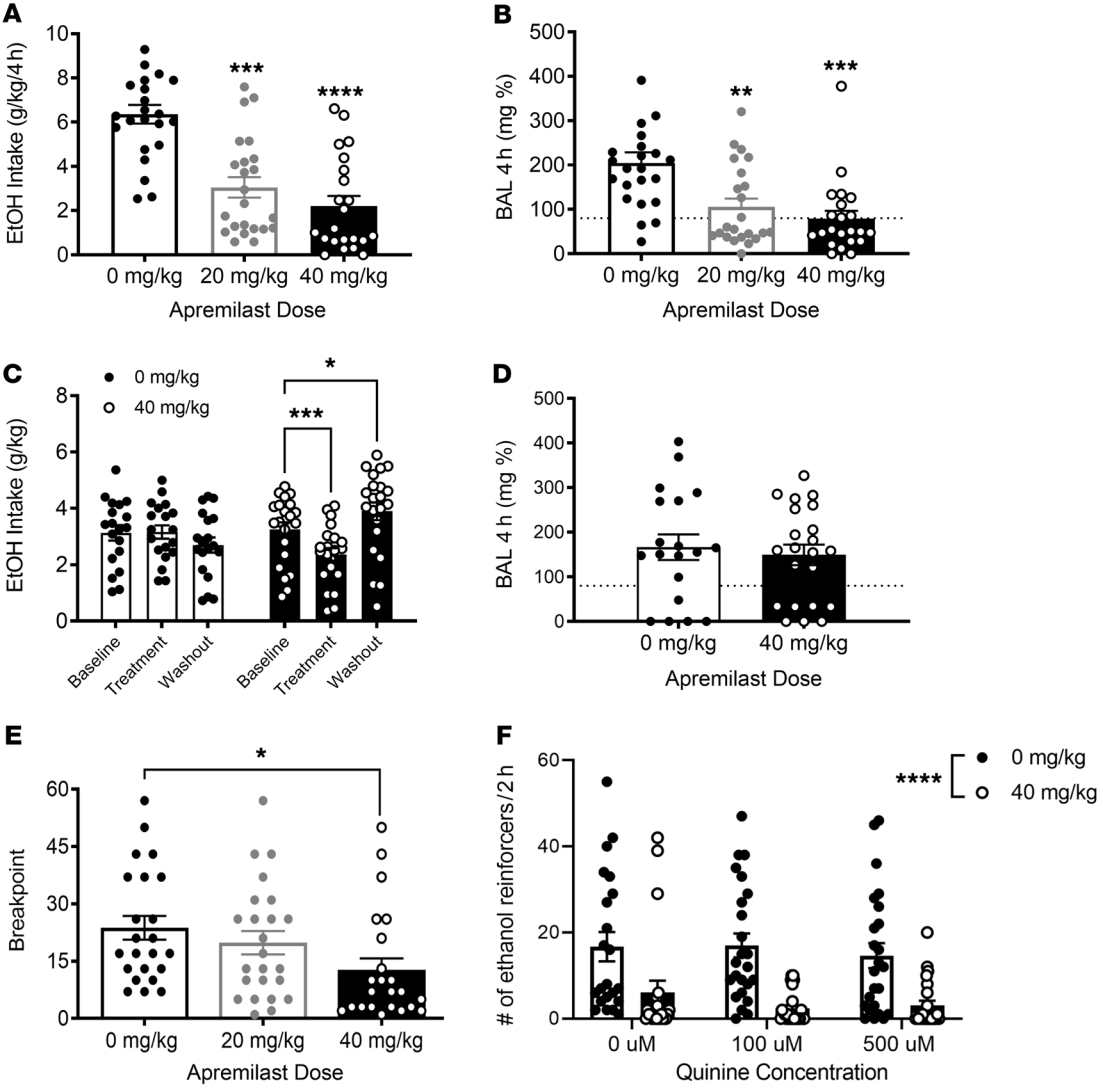
Apremilast reduces binge-like drinking behavior and ethanol motivation in mice selectively bred for drinking to intoxication. (**A**) Binge-like ethanol intake (g/kg/4 hours) for HDID-1 (*n* = 10–12/sex/apremilast dose; main effect of apremilast [*F*(2, 61) = 21.0, *P* < 0.0001], with no sex or sex × treatment interactions. Both doses of apremilast reduced ethanol (EtOH) intake in HDID-1 mice. (**B**) Blood alcohol levels (BALs, mg%) in HDID-1; main effect of apremilast [*F*(2, 64) = 9.73, *P* < 0.001]; both doses of apremilast reduced BALs compared with 0 mg/kg. (**C**) Average 4-hour ethanol intake over 6-week test (week 1, baseline; weeks 2–5, treatment; week 6, washout) for apremilast-treated HDID-1 mice (*n* = 10–12/sex/apremilast treatment); main effect of time [*F*(2, 78) = 5.68; *P* < 0.01] and a time × treatment interaction [*F*(2, 78) = 17.56; *P* < 0.0001]; 40 mg/kg reduced ethanol intake compared with baseline and washout intake was higher than baseline. (**D**) BALs (mg%) for end of week 5, 4-hour drinking; no effect of apremilast (2-tailed Student’s *t* test ). (**E**) Highest operant response ratio reached (breakpoint) during PR testing (marker of ethanol motivation) for iHDID-1 (*n* = 10/12/sex/apremilast treatment); main effect of treatment [*F*(2, 64) = 4.47; *P* < 0.05]; 40 mg/kg reduced breakpoint iHDID-1 mice. (**F**) Ethanol reinforcers earned during quinine-adulterated testing; main effect of apremilast treatment [*F*(1, 134) = 37.90; *P* < 0.0001], with no effect of quinine or apremilast × quinine interaction; 40 mg/kg apremilast reduced the number of reinforcers earned for iHDID-1 mice at all quinine concentrations tested. *P < 0.05; **P < 0.05; ***P < 0.001; ****P < 0.0001 by 2-way ANOVA followed by Dunnett’s post hoc test (**A**–**C**, **E**, and **F**).

Next, we found that 2 clinically relevant doses of apremilast, 20 and 40 mg/kg (i.p.) reduced binge drinking and BALs (below 80 mg% — the level of intoxication), in female and male HDID-1 mice (Figure 1, A and B). The ability of 2 different PDE4 inhibitor compounds to reduce drinking suggests that PDE4 is likely an important regulator of excessive alcohol drinking by animals with genetic risk and is consistent with prior studies of PDE4 inhibitors in other strains (23, 24). There was no effect of apremilast on water or saccharin intake, indicating the observed reduction in ethanol intake in HDID-1 mice was likely not due to sedation, sickness, or altered tastant reinforcement (Supplemental Figure 2, A and B). The same doses of apremilast reduced binge-like drinking and BALs in female and male HDID-2 mice (Supplemental Figure 3, A and B); however, there was an effect of apremilast on binge-like water and saccharin intake, suggesting that the reductions in alcohol drinking following apremilast treatment in HDID-2 mice may have resulted from general effects on liquid intake and/or malaise (Supplemental Figure 2, C and D). Similar differences in saccharin intake between HDID-1 and HDID-2 have been reported in earlier behavioral pharmacology findings (25, 26). Future work would benefit from addressing known differences in drinking microstructure and genetics between these replicate lines (27, 28).

AUDs are characterized by a chronic history of harmful drinking. To determine the efficacy of apremilast in reducing chronic alcohol intake, we tested whether 40 mg/kg (i.p.) of apremilast would reduce binge drinking in HDID-1 mice (of both sexes) over a 4-week period, as compared to baseline drinking levels. Here we saw that 40 mg/kg of apremilast reduced chronic binge-like ethanol intake of female and male HDID-1 mice (Figure 1C), with no effect on BALs (Figure 1D). Of note, we observed an increase in drinking after treatment ended (washout drinking levels; Figure 1 D). This suggests that termination of apremilast may lead to an increase in binge drinking.

To determine whether PDE4 inhibition reduces the motivation for alcohol drinking, we next tested the effects of apremilast in inbred HDID-1 (iHDID-1) mice of both sexes during 2 operant ethanol self-administration tasks: (a) operant responding under a progressive ratio (PR) schedule of reinforcement and (b) quinine-adulterated alcohol responding (9, 29, 30). Here we found that 40 mg/kg (i.p.) of apremilast reduced the total number of operant reinforcers earned during PR and the breakpoint (the highest response ratio reached; Figure 1E), and reduced intake during the PR test (Supplemental Figure 4C).

To ascertain whether apremilast would reduce compulsive-like responding to alcohol (another facet of human alcohol motivation), mice were tested for quinine-adulterated alcohol responding (see timeline in Supplemental Figure 4A) (10). Female and male iHDID-1 mice were given counterbalanced injections of 0 mg/kg and 40 mg/kg (i.p.) apremilast, prior to operant self-administration of quinine-adulterated ethanol (0, 100, and 500 μM). Apremilast reduced the number of alcohol access periods (reinforcers) earned and alcohol intake at all concentrations of quinine tested (Figure 1F and Supplemental Figure 4D). This suggests that apremilast reduced the motivation to drink despite negative consequences and taken together, these findings indicate that apremilast effectively reduces behavioral signs of alcohol motivation in mice bred to drink to intoxication.

### The NAc is a critical site of action for reduction of drinking by apremilast.

**Figure 2:**
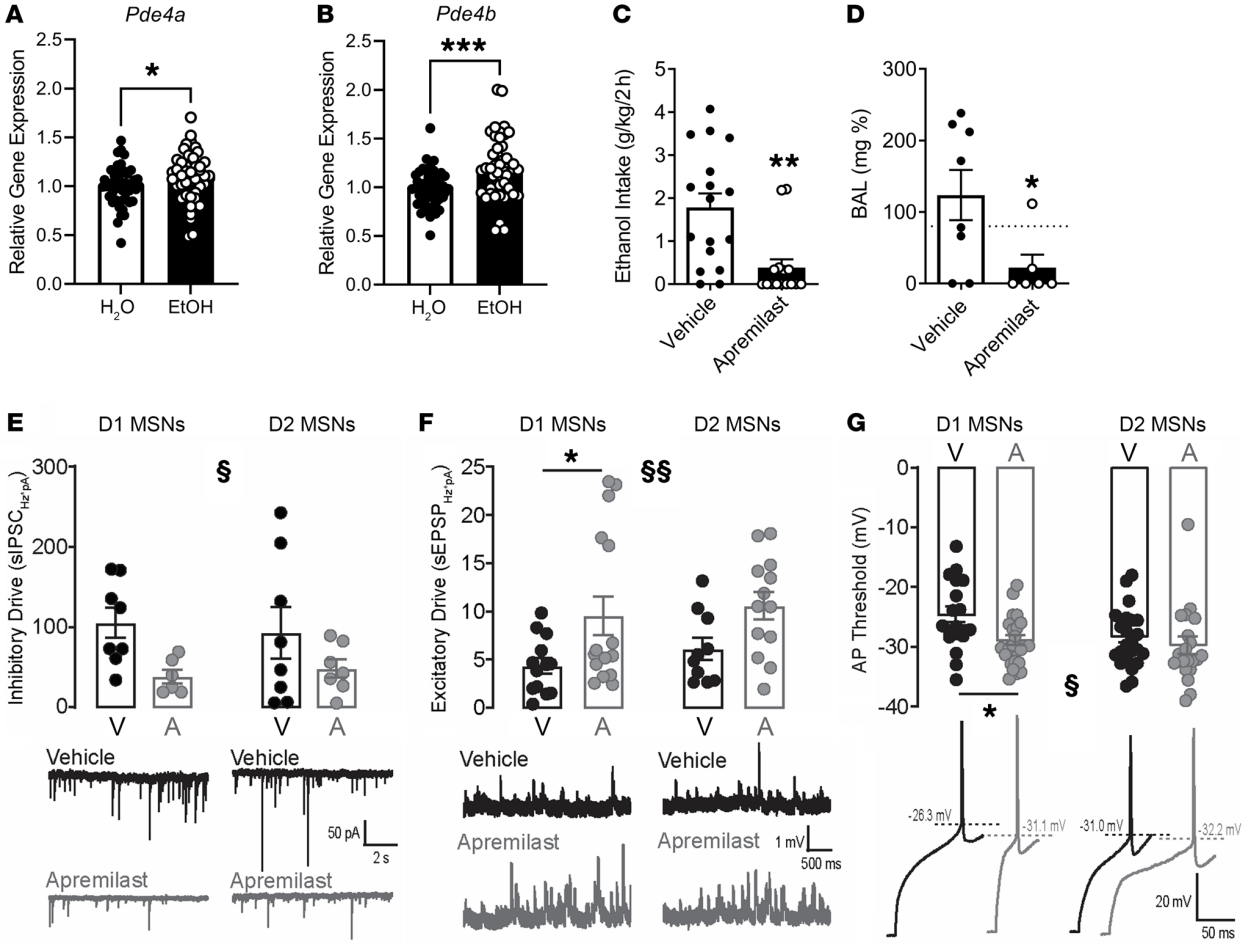
Apremilast reduces binge-like drinking behavior through increasing excitability of D1, but not D2, MSNs. (**A**) Relative NAc *Pde4a* gene expression for female HDID-1 mice (46–48/fluid group); significant effect of fluid type. Ethanol mice express higher levels of *PDE4a*. EtOH, ethanol. (**B**) Relative gene expression of NAc *Pde4b*; significant effect of fluid type. Ethanol mice express higher levels of *PDE4b*. (**C**) Ethanol intake (g/kg/2 hours) following intra-NAc apremilast infusions (0 or 2.2 μg/μL/side for male HDID-1 mice (*n* = 19–20/fluid group/infusion group) shows a significant effect of apremilast. (**D**) Blood alcohol levels (mg%); significant effect of apremilast. (**E**) Apremilast suppressed synaptic inhibition of NAc MSNs (*n* = 6–8/group). Inhibitory synaptic drive = frequency × current of spontaneous inhibitory postsynaptic currents (sIPSCs). § = main effect of treatment [*F*(1, 25) = 6.53, *P* < 0.05]. (**F**) Apremilast promoted synaptic excitation of NAc MSNs (*n* = 10–16/group). Excitatory synaptic drive = frequency × amplitude of spontaneous excitatory postsynaptic potentials (sEPSPs). §§ = main effect of treatment [*F*(1, 48) = 11.08, *P* < 0.01]. *P < 0.05, effect of treatment in D1 MSNs. (**G**) Apremilast promoted NAc output by lowering the threshold for MSN action potential (AP) firing (*n* = 19–24/group). § = main effect of treatment [*F*(1, 82) = 6.26, *P* < 0.05]. *P < 0.05, effect of treatment in D1 MSNs. V, vehicle (0.002% DMSO); A, apremilast (1 μM). Dashed lines indicate the AP threshold for each example trace. *P < 0.05, **P < 0.05, ***P < 0.001 by 2-tailed Student’s *t* test (**A**–**D**). Data in **E**–**G** were analyzed using 2- or 3-way ANOVA, with cell type (D1 or D2 MSN) and treatment condition (vehicle or apremilast) as between-groups factors. The effect of treatment within each MSN subtype was analyzed using Bonferroni’s multiple-comparison test.

Recent evidence suggests that increased expression of PDE4 subtypes, namely PDE4b, is linked to human AUD (31). Here we found that binge drinking increased the expression of both PDE4a and PDE4b subtypes in the NAc (a brain region integral to alcohol drinking) in HDID-1 mice (Figure 2, A and B). Therefore, to determine whether inhibition of PDE4 in the NAc could reduce drinking, we next tested the effects of intracranial NAc infusions of apremilast on binge drinking in HDID-1 mice. We observed a significant decrease in binge-like ethanol drinking and BALs (Figure 2, C and D), suggesting that PDE4 inhibition in the NAc alone is sufficient to reduce harmful drinking. Moreover, we saw no effect of apremilast on either water or saccharin intake, supporting the notion that the reduction was specific to ethanol and not total liquid intake or altered sensitivity to rewarding solutions (Supplemental Figure 5).

Next, we sought to determine how acute treatment with apremilast altered functional activity in NAc D1 MSNs and D2 MSNs, which together comprise greater than 90% of the neurons in the NAc and are the 2 major output pathways of this region. We performed ex vivo whole-cell patch clamp electrophysiology in brain slices from male and female hemizygous *Drd1a*-tdTomato mice and assessed the effects of apremilast on spontaneous synaptic activity (inhibitory postsynaptic currents and excitatory postsynaptic potentials) and cellular excitability. Inhibitory synaptic drive overall was suppressed by apremilast treatment (Figure 2E), and this effect was primarily mediated by a decreased frequency of spontaneous inhibitory postsynaptic currents in D1 MSNs (Supplemental Figure 6A). Excitatory synaptic drive overall, and in D1 MSNs in particular, was enhanced by apremilast treatment (Figure 2F and Supplemental Figure 6B). Apremilast also altered intrinsic excitability, particularly for D1 MSNs. Apremilast treatment produced a left shift in the input-output curve for evoked action potential firing by D1 MSNs, but had no such effect in D2 MSNs (Supplemental Figure 6C). The apremilast effect on evoked firing was not accompanied by treatment differences in membrane properties related to inwardly rectifying potassium channels (such as resting membrane potential, input resistance, or rheobase), or in action potential properties related to calcium-activated and voltage-gated potassium channels (such as the action potential half-width and afterhyperpolarization potential), which contribute to sustained firing capability (Supplemental Table 1). Rather, the action potential threshold of D1 MSNs was significantly reduced by apremilast treatment (Figure 2G), indicating voltage-gated sodium channels may be a downstream target of NAc PDE4 inhibition. Thus, in summary, these results indicate that apremilast treatment increases the net synaptic drive of both subtypes of NAc MSNs, but promotes NAc output primarily through D1 MSNs by increasing the responsiveness of these neurons to membrane depolarization.

Dopaminergic neurotransmission in NAc MSNs is largely mediated through PKA signaling, of which PDE4 is a critical regulator. Nishi et al. demonstrated that the PDE4 inhibitor rolipram increased neuronal excitability in isolated MSNs (32). There is evidence demonstrating that altering activity of the NAc leads to a decrease in alcohol craving and relapse in humans (19, 33, 34) and binge-like drinking in mice (20, 21, 35). Extending the importance of PDE4 inhibition to NAc-mediated ethanol drinking, the present findings show that site-specific apremilast treatment is sufficient to reduce binge-like ethanol drinking and may do so by promoting functional connectivity within efferent and afferent NAc pathways.

### Apremilast reduces dependence-induced escalations in alcohol intake in C57BL/6J mice.

**Figure 3:**
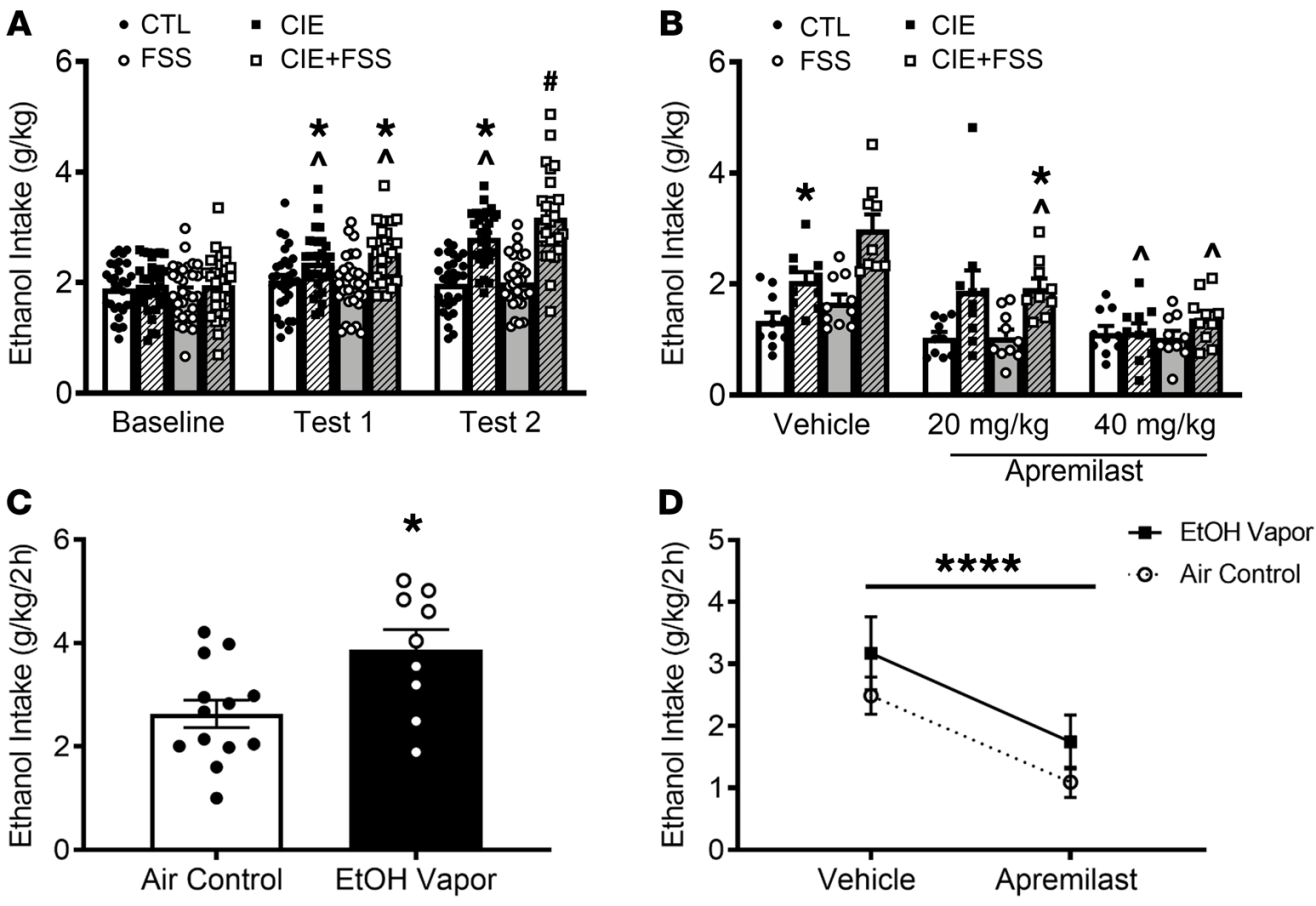
Apremilast reduces dependence-induced escalations in ethanol drinking in C57BL/6J mice. (**A**) Ethanol intake (g/kg/2 hours) for male C57BL/6J mice (*n* = 9–10/vapor group/stress group/apremilast treatment) during baseline and tests 1 and 2; main effect of group [*F*(3, 114) = 15.22; *P* < 0.001], phase [*F*(2, 114) = 60.80; *P* < 0.001], and a group × phase interaction [*F*(6, 228) = 13.25; *P* < 0.001]; CIE and CIE + FSS had higher intake compared with baseline and control (CTL) values (*P < 0.05) and their own baseline (^*P* < 0.05); CIE + FSS had higher intake in test 2 than all other groups and their own baseline (^#^*P* < 0.05). (**B**) Ethanol intake (g/kg/2 hours) during test 3; main effect of group [*F*(3, 106) = 16.28; *P* < 0.001], apremilast [*F*(2, 106) = 21.83; *P* < 0.001], and a group × treatment interaction [*F*(6, 106) = 3.25; *P* < 0.01]; for mice that received vehicle, ethanol intake was higher for CIE mice compared with CTL mice (*P < 0.05) and higher for CIE + FSS compared with the 3 groups that also received vehicle (^#^*P* < 0.05). CIE + FSS mice that received 20 mg/kg apremilast continued to drink more ethanol than CTL mice (*P < 0.05). However, this dose reduced ethanol intake compared with its vehicle condition group (^*P* < 0.05). The 40 mg/kg apremilast dose resulted in a significant decrease in ethanol intake in CIE and CIE + FSS mice compared with their vehicle equivalent (^*P* < 0.05). (**C**) Ethanol (EtOH) intake (g/kg/2 hours) for female and male C57BL/6J mice (*n* = 10/vapor group/apremilast treatment) following 3 weeks of CIE, main effect of vapor exposure, whereby ethanol vapor increased intake (*P < 0.05 by 1-way ANOVA). (**D**) Ethanol intake (g/kg/2 hours) during test week, main effect of treatment, 40 mg/kg (p.o.) reduced intake in ethanol vapor and air exposed mice. *P < 0.05, ***P < 0.001, ****P < 0.0001 by 2-way (**A** and **B**) or 1-way (**C** and **D**) ANOVA followed by Newman-Keuls post hoc test.

To test whether apremilast reduces harmful drinking associated with alcohol dependence, 2 models of dependence-induced escalations in ethanol drinking were used in C57BL/6J mice, an established high-drinking strain from which both methods were developed (13, 15). The experimental details and timelines are shown in Supplemental Figure 7. In the first set of experiments, a daily stressor (forced swim stress, FSS) was given in combination with chronic intermittent ethanol (CIE) vapor exposure to escalate drinking behavior (Figure 3A). Stress is thought to play a critical role in alcohol dependence, whereby FSS prior to CIE exposure has been shown to enhance escalation and alcohol intake beyond CIE alone (13, 14). Consistent with published findings, C57BL/6J mice exposed to CIE and those given stress in combination with CIE (CIE + FSS) had higher ethanol intake than air control mice and those given FSS alone (13) (Figure 3A). Here we found that 20 mg/kg of apremilast reduced alcohol intake in stressed, dependent (CIE + FSS) mice and that 40 mg/kg apremilast effectively reduced ethanol intake in stressed and nonstressed, dependent mice (Figure 3B).

**Figure 4:**
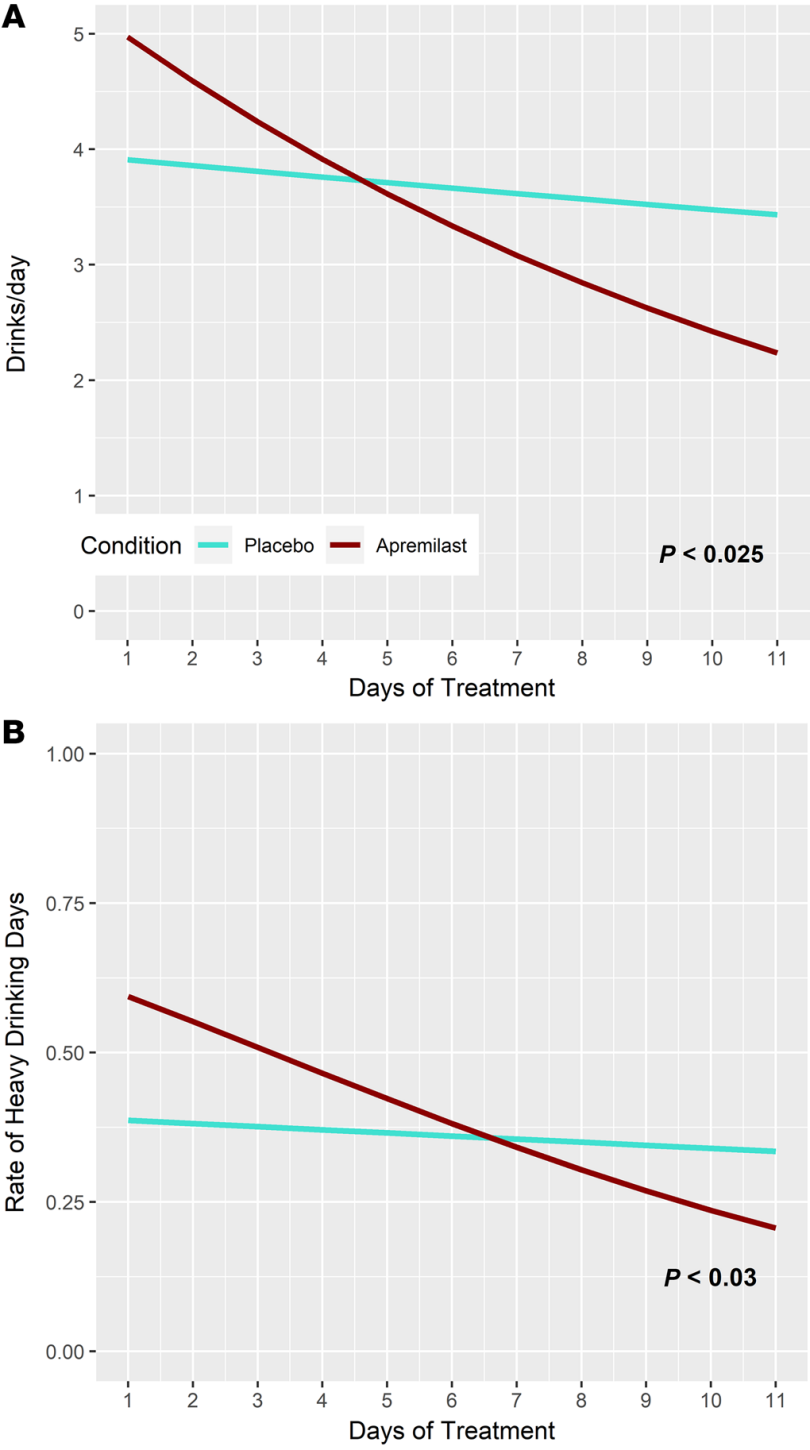
Apremilast reduces alcohol intake in non–treatment-seeking individuals with an AUD. (**A**) Apremilast (90 mg/d) significantly (*z* = 2.24; *P* < 0.025) reduces the number of drinks per day relative to placebo in 51 non–treatment-seeking individuals with alcohol use disorder of moderate severity or greater. A negative binomial latent growth curve model was used to calculate an effect size for apremilast versus placebo in the decrease in drinks per day from baseline through 11 days of ad libitum drinking. This procedure generated 11-day change values totaling 2.74 drinks per day for apremilast and 0.48 for placebo, and yields a Cohen’s *d* value of 0.77, which can be interpreted as a “large” effect of apremilast on decreasing drinking. (**B**) Proportion of heavy drinking days (4+ for women, 5+ for men) is significantly (*z* = 2.17, *P* = 0.030) reduced for apremilast versus placebo. A latent logistic regression model (retransformed to units of proportion) was used to calculate differences in daily risk of heavy drinking through 11 days of treatment. Risk reduction between day 1 and day 11 was 0.39 for apremilast versus 0.05 for placebo; Cohen’s *d* for this difference was 0.26, a “small-medium” value.

Because dependence in individuals with AUD is characterized by chronic harmful drinking, we next sought to test the effects of apremilast in a chronic model of dependence-induced escalations in alcohol drinking (Supplemental Figure 7B). Here, female and male C57BL/6J mice underwent a standard CIE protocol (15, 36–39). Following 4 cycles of ethanol vapor exposure, mice showed an increase in alcohol intake relative to control mice (Figure 3C). When given orally prior to the last day of drinking, apremilast (20 mg/kg) was shown to decrease ethanol intake in nondependent (air control) and dependent mice (Figure 4D). In all, the above findings extend the efficacy of apremilast to reduce excessive ethanol drinking in 2 well-established animal models of alcohol dependence.

### Individuals with AUD consume fewer drinks per day when treated with apremilast.

A phase IIa double-blind, placebo-controlled POC study was conducted with the aim of clinically validating the effect of apremilast on decreasing alcohol intake in preclinical models of AUD. It was hypothesized that individuals with AUD who were treated with apremilast would consume significantly fewer standard drinks (~14 grams of alcohol per drink) per day over an 11-day period of ad libitum drinking than those treated with placebo. To further clarify whether any such effect was a result of reduction in heavy drinking specifically, numbers of heavy drinking days (4+ drinks/day for women, 5+ drinks/day for men) were similarly examined over this same period. Earlier PDE4 inhibitors like rolipram and ibudilast are associated with side effects, particularly nausea and vomiting, that may significantly reduce treatment retention (40). Apremilast shows less severe PDE4 adverse reactions (41). Thus, it may be well tolerated in the 50% higher than standard dose that is indicated for reducing drinking in AUD, using the above animal data to estimate human dose equivalence.

Study admission criteria specified non–treatment-seeking male and female paid volunteers 18 to 65 years of age with AUD of moderately severe or greater, as defined by the Diagnostic and Statistical Manual for Mental Disorders – Fifth Edition (DSM-5) criteria (42). The Consolidated Standards of Reporting Trials (CONSORT) flow diagram is shown in Supplemental Figure 8. Participants were randomly assigned in a 1:1 ratio to treatment with a target dose of 90 mg/d of apremilast or matched placebo in a parallel-group design. The randomization code included stratification on sex and baseline C-reactive protein (CRP; a blood marker of inflammation analyzed by LabCorp, <2 mg/L vs. ≥2 mg/L) status to ensure an equivalent distribution of participants across groups on 2 factors potentially related to outcome (Supplemental Table 2). Plasma levels of cytokines (TNF-α, CCL2, CXXL10), cortisol, apremilast, and serum endotoxin were assayed after study completion for evaluation as potential physiological moderators of treatment response (Supplemental Table 3).

The rate of study completion (84%) was equivalent across groups and is detailed in Supplemental Figure 8. Participants consisted of 24 (47.1%) females and 27 (52.9%) males, with a mean age of 41.2 (±16.3) years. Participants had been drinking heavily for 12.3 (±10.5) years and met criteria for 6.4 (±2.3) DSM-5 symptoms at baseline, indicating a severe level of AUD. Apremilast (*n* = 26) and placebo (*n* = 25) groups did not differ in baseline demographic, clinical, or physiological variables, as summarized in Supplemental Tables 2 and 3. Likewise, pretreatment drinks per day, rate of heavy drinking, and number of DSM-5 symptoms did not differ significantly between study finishers and dropouts (*t* test *P* values were all >0.40).

All participants completing the protocol (*n* = 43) were included in hypothesis testing that employed latent growth modeling (LGM) (43) to compare means and trends in daily drinking in apremilast versus placebo groups during the 11-day period of ad libitum drinking (R package glmmadmb v0.8.3.3) (44). Apremilast significantly (*P* < 0.05) reduced the number of drinks per day relative to placebo, as well as the probability of a heavy drinking day (*P* = 0.030; Figure 4, A and B). The associated LGM results are given in Supplemental Tables 4 and 5. Supplemental Table 4 shows a greater decline in drinking for the apremilast group versus placebo (*β* = –0.669, *z* = 2.24, *P* = 0.025). Calculations from the LGM show an average change from day 1 to day 11 of 2.74 drinks per day for apremilast and 0.48 for placebo and yields a Cohen’s *d* value of 0.77, which is consistent with a “large” effect of apremilast on decreasing drinking (45). Supplemental Table 5 shows that the log-odds of a heavy drinking day also declined more in the apremilast group versus placebo (*β* = –0.1504, *z* = 2.17, *P* = 0.030). The analogously calculated effect size of treatment was 0.39 for apremilast and 0.05 for placebo, with a Cohen’s *d* of 0.26, or “small-medium,” suggesting that some but not all of the decrease in drinking involved attenuation of heavy drinking. There was no evidence of rebound drinking differences in the 2 groups during the 2-week posttreatment follow-up period. No baseline demographic, clinical, or physiological variable contributed significantly to drinking outcome. A trend was noted (*P* < 0.09) that individuals in the apremilast group with higher baseline craving scores reduced their drinking at a faster rate than placebo (results not shown), and subjective reports also referenced decreased craving (Supplemental Table 6). No serious or severe adverse events occurred. Although adverse drug effects — diarrhea, nausea, abdominal pain, and somnolence — were 2 or more times more likely with apremilast than placebo, these effects were typically mild and were not associated with treatment discontinuation (Supplemental Table 7 and Supplemental Figure 8).

## Discussion

We leveraged gene expression profiles of drinking to intoxication to identify compounds that might be repurposed to reduce excessive alcohol drinking characteristic of AUD. The FDA-approved PDE4 inhibitor apremilast was identified as the most promising target for repurposing, given a lower likelihood of severe PDE4 adverse effects associated with treatment discontinuation than earlier PDE4 inhibitors. We propose it is imperative to test potential therapeutics across multiple drinking paradigms, species, and strains to reduce the number of clinical study failures. The present work determined whether apremilast would reduce harmful alcohol drinking in male and female mice from 4 different strains with high risk for excessive drinking (i.e., selectively bred HDID-1 and 2, inbred HDID-1 mice, and C57BL/6J mice). Strikingly, we found that apremilast reduced excessive drinking across a spectrum of clinically relevant drinking models for binge-like, motivational, compulsive-like, and stress- and non–stress-induced facilitation of dependence-like drinking. Although follow-up testing suggests that apremilast acts through central means (i.e., the NAc), it is possible that either or both central and peripheral actions of apremilast are necessary for reducing harmful drinking. Therefore, future work should address the importance of such central and peripheral mechanisms. In a human POC study, we employed a double-blind, placebo-controlled study in non–treatment-seeking individuals with AUD and found that oral apremilast was robustly effective at reducing the number of daily drinks consumed. PDE4b, a target of apremilast, has been associated with both alcohol and nicotine dependence. We show that a gene identified from a genome-wide association study (7), and a compound targeting its gene product identified from a separate transcriptomics study (46), successfully reduced harmful alcohol drinking across preclinical models of AUD and in humans with AUD. The approaches we used offer researchers studying complex diseases renewed opportunities to discover new or repurpose existing compounds and expedite treatment options.

Because apremilast works across a spectrum of models, in both sexes of 4 strains of mice (at multiple labs and universities) and importantly, in humans, we sought to determine the neural mechanisms by which PDE4 inhibition reduces harmful drinking. The NAc is a critical brain region for many behaviors and is well studied for its role in alcohol drinking. Structural and molecular changes in the NAc following both acute and chronic ethanol drinking are thought to play a role in further aberrant drinking patterns (47). Deep brain stimulation of the NAc decreases alcohol craving and relapse in humans (19, 33) and reduces alcohol drinking in rodents (48, 49). The findings herein show that chronic binge drinking results in increased NAc expression of 2 *Pde4* subtypes, *Pde4a* and *Pde4b*. Notably, heightened expression of the *Pde4b* isoform has been genetically associated with chronic ethanol intake in humans (31). We further found that apremilast’s effects at the level of the NAc are critical for reducing excessive drinking and for regulating neuronal activity in specific cell types of a neural circuit relevant to alcohol-related behaviors (50, 51). PDE4 inhibition has been shown to increase pre- and postsynaptic cAMP-driven markers of neuronal excitability in the NAc (32). PDE inhibitors are known to have effects on several signaling pathways, many of which may lead to altered neural function and changes in behavior (52). The present findings show that site-specific apremilast treatment is sufficient to reduce binge-like alcohol drinking, demonstrating the importance of PDE4 inhibition in the NAc for reducing drinking.

The extent to which PDE4 inhibition, and in particular apremilast, alters input to, and output of, subpopulations of MSNs in the NAc helps to identify potential critical neurobiological mechanisms and may in part explain the observed reduction in harmful alcohol drinking across drinking models. Decreased alcohol drinking seen with chemogenetic manipulation of the NAc likely engages different signaling pathways that may be dependent on drinking paradigm, sex, and/or distinct cell types (10, 20, 35, 53, 54). Here, we saw that apremilast treatment regulated both excitatory and inhibitory synaptic inputs to NAc MSNs overall, and the net consequence for NAc output was increased excitability of D1-, but not D2-expressing, MSNs. Our experiments do not definitively establish whether these electrophysiological responses to apremilast are necessary for its efficacy, but our electrophysiology findings, when considered alongside our observation that intra-NAc apremilast was sufficient to reduce alcohol intake, do serve to identify regulation of NAc MSN functional activity as a putative mechanism of action through which apremilast regulates alcohol-related behaviors.

The above double-blind, placebo-controlled POC study found a large effect of apremilast (90 mg/d) on decreasing drinking relative to placebo in 51 non–treatment-seeking men and women with severe AUD. The observed effect size for apremilast was more than double that reported in a comprehensive meta-analysis of randomized controlled trials of the FDA-approved treatments for AUD, acamprosate and naltrexone (55). Ibudilast, an older, less selective PDE inhibitor (targets PDE3, PDE4, PDE10, and PDE11), has been shown to reduce drinking in alcohol-dependent mice (CIE), but not nondependent mice (56). Ibudilast did not reduce the overall probability of drinking in a clinical POC study, but did reduce the number of heavy drinking days in individuals with AUD (57). Moreover, recent evidence suggests that ibudilast may modulate mood-dependent alcohol craving (58). Thus, our data offer support for a more selective compound with greater efficacy across a spectrum of AUD severity and outcomes. A somewhat smaller but still statistically significant effect of apremilast relative to placebo was found for reduction in risk of having a heavy drinking day. Together, these findings suggest apremilast reduces both the daily quantity of alcohol consumed as well as the frequency of heavy drinking episodes. Results provide clinical validation of PDE4 inhibition as a general therapeutic strategy for AUD, and specifically for our extensive preclinical data showing apremilast decreases drinking in animal models of AUD. The 90 mg/d dose, while 50% higher than standard dosing for psoriasis, was well tolerated in this AUD sample and was not associated with adverse events resulting in treatment discontinuation. This evidence of drug tolerability, combined with an association between reduced drinking and craving, suggests an anticraving effect of apremilast in reducing drinking, as opposed to an effect of adverse drug experiences. Taken together, these POC efficacy and safety data lend support to further development of apremilast as a treatment for AUD.

This collaborative set of studies from 6 independent laboratories and universities highlights apremilast as a powerful AUD treatment option and further identifies mechanisms by which apremilast may reduce harmful alcohol drinking.

## Methods

Further information can be found in Supplemental Methods.

### Data availability.

The drinking, behavioral, gene expression, and electrophysiological data supporting the findings of this study have been deposited and are available in the Figshare digital repository (10.6084/m9.figshare.14687358).

### Statistics.

Significance was set at an *α* value of 0.05. Behavioral experiments were analyzed with either a 1- or 2-way ANOVA, followed by either Dunnett’s or Newman-Keuls post hoc test. Two-tailed Student’s *t* test was used to analyze data from the intra-NAc apremilast and NAc mRNA studies. For electrophysiology experiments, data were analyzed using 2- or 3-way ANOVA. Changes in drinks per day and the probability of a heavy drinking day in the clinical study were analyzed using 2-tailed mixed-effect LGMs.

### Study approvals.

For animal studies, all procedures were approved by the local Institutional Animal Care and Use Committee and were conducted in accordance with the NIH *Guide for the Care and Use of Laboratory Animals* (National Academies Press, 2011). Human laboratory testing was approved by the Scripps Research Institutional Review Board (protocol 16-6821), was conducted under an investigator-initiated IND (no. 135813), and is registered on ClinicalTrials.gov (NCT03175549). All participants provided written informed consent prior to study inclusion.

## Author contributions

Preclinical: ARO, KBG, RA Mangieri, RA Morrisett, AJR, and MFL conceived and performed the preclinical experiments, performed analyses, and wrote the manuscript. EJF, AT, KGT, and HCA performed the preclinical experiments. PM analyzed preclinical data. JCC, MR, and HCB conceived the preclinical experiments and edited the manuscript. Clinical: BJM conceived and conducted the clinical study, interpreted results, and wrote the manuscript. JML analyzed the clinical data, interpreted results, and wrote the manuscript. AB, JB, JM, SQ, FS, and MS conducted the clinical study. TKE and JJM analyzed the physiological data and interpreted results.

## Supplementary Material

Supplemental data

## Version 1 01/19/2023

In-Press Preview

## Version 2 03/15/2023

Electronic publication
